# Molecular Identification of a Newly Isolated *Bacillus subtilis* BI19 and Optimization of Production Conditions for Enhanced Production of Extracellular Amylase

**DOI:** 10.1155/2015/859805

**Published:** 2015-06-09

**Authors:** Biplab Kumar Dash, M. Mizanur Rahman, Palash Kumar Sarker

**Affiliations:** ^1^Department of Biotechnology and Genetic Engineering, Faculty of Applied Science and Technology, Islamic University, Kushtia 7003, Bangladesh; ^2^Department of Genetic Engineering and Biotechnology, Faculty of Biological Science and Technology, Jessore University of Science and Technology, Jessore 7408, Bangladesh; ^3^Microbial Biotechnology Division, National Institute of Biotechnology, Savar, Dhaka 1349, Bangladesh

## Abstract

A study was carried out with a newly isolated bacterial strain yielding extracellular amylase. The phylogenetic tree constructed on the basis of 16S rDNA gene sequences revealed this strain as clustered with the closest members of *Bacillus* sp. and identified as *Bacillus subtilis* BI19. The effect of various fermentation conditions on amylase production through shake-flask culture was investigated. Rice flour (1.25%) as a cheap natural carbon source was found to induce amylase production mostly. A combination of peptone and tryptone as organic and ammonium sulfate as inorganic nitrogen sources gave highest yield. Maximum production was obtained after 24 h of incubation at 37°C with an initial medium pH 8.0. Addition of surfactants like Tween 80 (0.25 g/L) and sodium lauryl sulfate (0.2 g/L) resulted in 28% and 15% increase in enzyme production, respectively. Amylase production was 3.06 times higher when optimized production conditions were used. Optimum reaction temperature and pH for crude amylase activity were 50°C and 6.0, respectively. The crude enzyme showed activity and stability over a fair range of temperature and pH. These results suggest that *B. subtilis* BI19 could be exploited for production of amylase at relatively low cost and time.

## 1. Background

Amylase represents a group of extracellular enzymes (consisting of *α*-amylase, *β*-amylase, and glucoamylase) that act on starch or oligosaccharide molecules in a random manner and hydrolyze into diverse products including dextrins and progressively smaller polymers composed of glucose units [1]. They have most widely been reported to occur in micro-organisms (fungi, yeast, bacteria, and actinomycetes), although they are found in plants and animals [2]. In present day they have found applications in all the industrial processes such as in food, detergents, textiles, pharmaceutical, paper and fine chemical industries for the hydrolysis of starch [3–5]. Amylase has great significance in present-day biotechnology having approximately 25–30% of the world enzyme market [6]. These extensive potentials of amylase to be used in broad range of industries have placed greater stress on researchers to search for more efficient amylase production.

The genus* Bacillus *has been becoming a reliable option to find out novel and promising bacteria for the production of amylase and other extracellular enzymes. Different species of* Bacillus*, most notably,* B*.* subtilis*,* B*.* licheniformis, B*.* amyloliquefaciens*, and* B*.* stearothermophilus*, are reported to produce approximately 60% of commercially available enzymes [7]. Short fermentation cycle, capacity to secrete proteins into the extracellular medium, safe handling, eco-friendly behavior, easy manipulation to obtain enzymes of desired characteristics, high enzymatic activity in a wide range of conditions (extreme pH, temperature, osmolarity, pressure, etc.), and simple and cost effective production have made this genus as bacterial workhorses for the production of a variety of enzymes as well as fine biochemicals for decades [8, 9]. Different* Bacillus *species have similar growth patterns and enzyme profiles but depending upon the strain their general properties (thermostability, pH profile, pH stability, etc.) and optimized fermentation conditions may vary [10]. Thus it is really challenging to obtain a strain that can produce amylase meeting specific industrial demands.

There is no local production and thereby availability of amylase in Bangladesh. As a result, most of the existing and growing starch based industries are using expensive chemicals for starch hydrolysing based purposes. Keeping in mind the growing demand of amylases by different industrial sectors this study was carried out to obtain laboratory scale fermentation of amylases in shake flask culture by newly isolated* B. subtilis* BI19 along with optimization of medium components and culture conditions for enhanced production, thereby to understand its potential for biotechnological application.

## 2. Materials and Methods

### 2.1. Isolation and Screening of Amylolytic of Bacteria

The soil samples were collected from different areas of Savar, Dhaka, in the month of August, 2011. Bacteria were isolated by serial dilution and spread plate method in nutrient agar (NA) (Oxoid, UK). Before spreading diluted soil samples were heated at 90°C for 15 min. Isolated pure cultures were primarily screened for amylase activity by employing zone clearing technique on starch agar plate containing 1% starch (BDH, England) fortified with NA [11]. Then they were assessed for potency index (PI) according to Ball and McGonagle (1978) [12]. Higher PI value indicates the greater ability of an isolate to produce extracellular enzyme [13].

### 2.2. 16S rDNA Sequence Analysis for Identification of Bacteria

DNA was extracted from single colony by alkaline lysis [14, 15]. Extracted DNA was stored at −20°C for further molecular analyses. 16S rDNA amplification and sequencing was performed as described by Rahman et al. (2014a, b) [14, 15]. Primers used to amplify 16S rDNA sequence were forward: 63F 5′CAGGCCTAACACATGCAAGTC [16] and reverse: 1389R 5′ACGGGCGGTGTGTACAAG [17] in a PCR thermal cycler (ICycler 170-8740, USA). The amplified DNA was visualized by gel electrophoresis and sequenced. The 16S rDNA sequence was analyzed using Chromas LITE (Version 2.01); the most similar bacterial species was found in the GenBank by using BLAST search (http://www.ncbi.nlm.nih.gov/). Neighbor-joining phylogenetic trees were constructed based on 16S rDNA sequences using ClustalW.

### 2.3. Preparation of Inoculum

Vegetative inoculums were used in the present studies. Fifty (50) mL of inoculum medium containing nutrient broth 13 g/L, pH 7.4, was transferred to a 250 mL Erlenmeyer flask and was sterilized in an autoclave (CL-40M, Japan) at 15 lbs/inch^2^ pressure at 121°C for 20 min. After cooling at room temperature, a loopful of freshly grown bacterial culture was aseptically transferred to it. The flask was incubated overnight at 37°C and 150 rpm in a rotary shaking incubator (Stuart SI 500, UK).

### 2.4. Submerged Fermentation for Amylase Production

Amylase production was carried out in basal medium containing 1.0% starch, 1% peptone, 0.8% (NH_4_)_2_SO_4_, 0.2% MgSO_4_·7H_2_O, 0.05% CaCl_2_·2H_2_O, 1.4% K_2_HPO_4_, and 0.6% KH_2_PO_4_ (a slight modification of Sarikaya and Gürgün, 2000; a single nitrogenous source instead of two was used) [18]. One (1) mL (2%) of 24 h grown inoculums was cultivated in 250 mL Erlenmeyer flasks containing 50 mL (w/v) of medium with an initial pH 7.0. The cultures were shaken at 150 rpm in an orbital shaker incubator at 37°C for at least 72 h unless otherwise stated. After incubation, fermented broth was centrifuged in a refrigerated centrifuge machine (Hitachi CF16RXII, Japan) at 8000 rpm for 15 min at 4°C. Cell free supernatant was collected and preserved for the estimation of amylase activity. To optimize the medium components various carbon sources, organic and inorganic nitrogen sources, and added surfactants and polyhydroxy alcohols were varied in different concentrations in the basal medium one at a time while other ingredients were kept constant.

### 2.5. Enzyme Assay

Amylase was determined by using soluble starch, 1% (w/v), as substrate in 0.05 M sodium phosphate buffer (pH 6.5) essentially according to Gomes et al. (2001) [19]. The reaction mixture containing 1.8 mL substrate solution and 0.2 mL suitably diluted enzyme solution was incubated at 50°C for 10 min. The reaction was stopped by adding 3 mL dinitrosalicylic acid (DNS). The reducing sugar released was determined by the method of Miller (1959) [20]. The absorbance was measured at 540 nm with spectrophotometer (Jenway 6305, USA). One unit (U) of enzyme activity is defined in all cases as the amount of enzyme releasing 1 *μ*g of reducing sugar as maltose per minute, under assay conditions.

(1) $$\begin{array}{c} \text{Enzyme}\;\text{Activity}\;\left(\text{U}/\text{mL}/\min\right)=\frac{\text{Sugar}\;\text{released}\;\left(µ\text{g}\right)\times \text{Total}\;\text{volume}\;\text{of}\;\text{reactive}\;\text{media}\;\left(\text{mL}\right)\times \text{Dilution}\;\text{factor}\;\left(\text{DF}\right)}{\text{Molecular}\;\text{weight}\;\text{of}\;\text{maltose}\times \text{Enzyme}\;\text{used}\;\left(\text{mL}\right)\times \text{Time}\;\text{of}\;\text{incubation}\;\left(\min\right)}. \end{array}$$

### 2.6. Partial Characterization of Crude Amylase

Enzyme samples were incubated for 10 min at temperatures ranging from 30 to 90°C in 0.05 M sodium phosphate buffer (pH 6.5). Thermal stabilities were determined by heating enzyme without the substrate fractions at various temperatures between 30 and 60°C for 1 h. At 10 min intervals, aliquots of 1 mL of the incubated enzyme were assayed for activity. The optimum pH for the enzyme activity was determined in different pH (4.0–9.0). The pH stability was determined by incubating the enzyme in 0.05 M sodium phosphate buffer (pH 6.5) with different pH values for 2 h at room temperature (25°C).

### 2.7. Statistical Analysis

A statistical package (SPSS version 11.0, SPSS Inc., Chicago, IL) was used for the data analysis. Each experiment was run in triplicate. Mean values and standard deviations were calculated.

## 3. Results and Discussion

### 3.1. Isolation, Screening, and Identification of Amylolytic Bacteria

Isolation and selection of suitable organism are essential for the production of extracellular amylases. Members of genus* Bacillus *were found to be better producer of different types of amylase [21, 22]. In this connection, a total of 35 morphologically well-formed single colonies were selected from different soil samples on the basis of their morphological differences in NA plates. Among them 19 strains were found to be positive as amylase producers. Finally, BI19 strain was selected as the best amylase producer according to highest potency index value. On the basis of multiple sequence alignments to rooted phylogenic tree with branch length (UPGMA) of 16S rDNA sequence by CLUSTALW, the strain BI19 exhibited high level (99%) of similarity with the known sequences in the public databases in NCBI and BLAST results and identified as* Bacillus subtilis *(accession number FJ527663).

### 3.2. Optimization of Carbon Sources, Organic Nitrogenous, and Inorganic Nitrogenous Sources for Amylase Production

The cell growth and production of amylase by* Bacillus *sp. is reported to be dependent on the strain, composition, and concentration of media, methods of cultivation, cell growth, nutrient requirements, pH, temperature, time of incubation, and thermostability [22, 23]. Thus to enhance the final production level it is essential to screen various medium components and cultural conditions associated with the growth of the inoculum [24].

**Figure 1:**
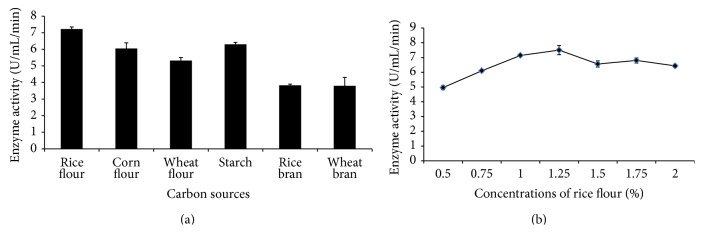
The production of amylase by* Bacillus subtilis *BI19 in shake flask fermentation. (a) Effect of different carbon sources and (b) effect of different concentrations of rice flour. Each value is an average of three replicates, pH 7.0, incubation period 72 h, incubation temperature 37°C, agitation speed 150 rpm, and inoculums volume 2%.

The production of amylase by* B. subtilis* reported to be effected by various carbon sources [5]. In our study, rice flour, starch and corn flour were found to be the stimulator of amylase production (Figure 1(a)). It may be due to starch and rice flour metabolized slowly by the bacterium as complex carbohydrate sources with increasing accumulation of inducible amylase in the fermentation medium [25]. Rice flour at a concentration of 1.25% (w/v) supported optimal enzyme production, followed by a decline at higher concentrations (Figure 1(b)). This can be attributed to the high viscosity of culture broth at such concentrations that interferes with O_2_ transfer leading to limitations of dissolved O_2_ for growth of bacteria [26]. Hence, these starch-rich rice and corn flours may prove useful and cheaper alternative natural sources of carbon and energy for the bacterial production of amylase.

**Figure 2:**
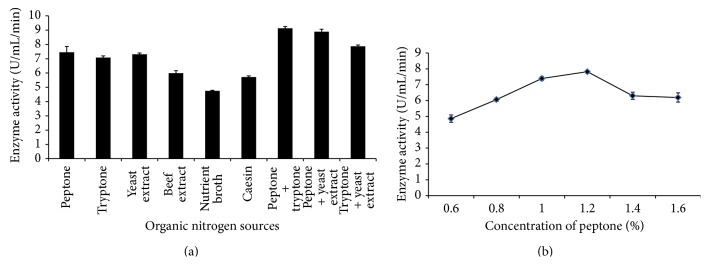
The production of amylase by* Bacillus subtilis *BI19 in shake flask fermentation. (a) Effect of different organic nitrogen sources and (b) effect of different concentrations of peptone.

**Figure 3:**
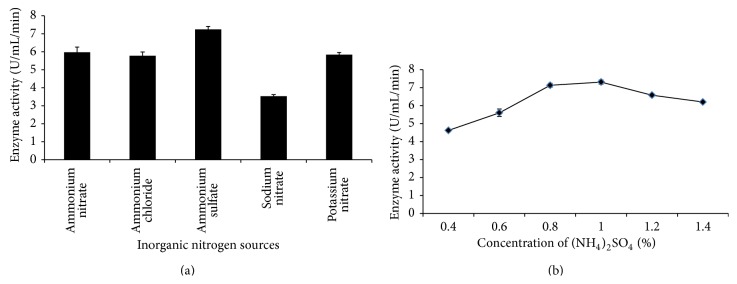
The production of amylase by* Bacillus subtilis *BI19 in shake flask fermentation. (a) Effect of different inorganic nitrogen sources and (b) effect of different concentrations of ammonium sulfate.

The nature and relative concentration of different complex nitrogenous sources in the growth medium are both important in the synthesis of amylase. Like lower levels, higher levels of nitrogen are equally detrimental causing enzyme inhibition [27]. Various complex nitrogen sources were added separately and in combination as replacement of peptone (1%) to the original medium to assess their effects on the final production (Figure 2(a)). It has been previously found that organic nitrogen sources like peptone and yeast extract usually have stimulating effects [28] and our findings are similar to them. Yeast extract also reported to serve as good organic nitrogen source for *α*-amylase synthesis from* B. amyloliquefaciens* [27]. Bozic et al. (2011) [29] found casein to be the best nitrogen source for *α*-amylase production from* B. subtilis* IP 5832. Albeit peptone as single replacement was significant; all the combination of peptone, tryptone, and yeast extract gave better results for amylase production in this experiment. Nusrat and Rahman (2007) [21] reported similar results for *α*-amylase production by* B. licheniformis *and* B. subtilis*. As a single organic nitrogen source, 1.2% of peptone was found to produce maximum amylase (7.82 U/mL/min) (Figure 2(b)). Inorganic nitrogen sources likely ammonium salts have been reported to induce amylase production [4]. Our findings are in good agreement with these studies. Presence of 1% (NH_4_)_2_SO_4_ was found to give maximum yield (7.31 U/mL/min) of amylase in this experiment (Figures 3(a) and 3(b)). The decline in amylase production at increased nitrogen concentration could be due to the lowering of pH of the production medium or induction of protease, which suppresses the amylolytic activity [23]. Swain et al. (2006) [30] reported to find suppressed *α*-amylase production by newly isolated* B. subtilis* when 1% ammonium sulphate was used in the fermentation medium which is contrary with our findings.

### 3.3. Effect of Added Glucose, Surfactants, and Polyhydroxy Alcohols on Production of Amylase

**Figure 4:**
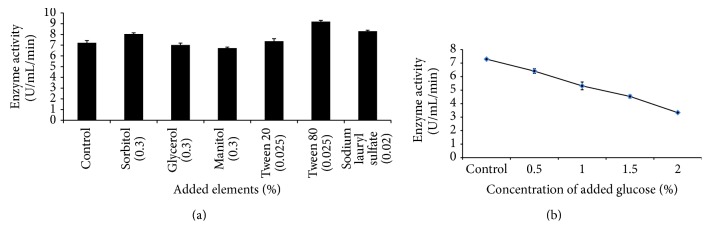
The production of amylase by* Bacillus subtilis *BI19 in shake flask fermentation. (a) Effect of addition of different concentrations of glucose in basal medium and (b) effect of addition of various elements in basal medium.

Addition of free glucose in the fermentation medium was found to suppress amylase production greatly as shown in Figure 4(a). The inhibitory effect of glucose on *α*-amylase synthesis increased with the increase of glucose concentration in the medium. Addition of 2% glucose resulted in about 54% of production loss (3.33 U/mL/min). Similar results were found by Nusrat and Rahman (2007) [21]. The most possible reason may the suppression of synthesis of carbohydrate degrading enzymes by readily metabolizable substrates such as glucose and fructose (mediated by the protein encoded by the CreA gene) [31].

Addition of surfactants and polyhydroxy alcohols in production medium reported to increase amylase secretion [32]. In this study, amylase production was found to increase by 28%, 15%, and 12%, respectively, in culture medium over control due to addition of Tween 80 (0.025%), sodium lauryl sulfate (0.02%), and sorbitol (0.3%) whereas glycerol (0.3%) and mannitol (0.3%) were found to suppress the production (Figure 4(b)). This increase in production might be due to increase in cell membrane permeability [33] and/or modification (swelling) of starch [34]. Palit and Banerjee (2001) [35] found the similar result with* B. circulans*.

### 3.4. Optimization of Incubation Temperature, Initial Medium pH, Incubation Period, and Agitation Speed for Amylase Production

The influence of temperature on amylase production is related to the growth of the organism. The temperature range of 35–45°C reported to be preferred for the biosynthesis of amylases by* Bacillus *strains [3, 4].* B*.* amyloliquefaciens*,* B*.* subtilis*,* B*.* licheniformis, *and* B*.* stearothermophilus *are among the most commonly used* Bacillus *sp. reported to produce amylase at temperatures 37–60°C [36]. In the present study, production of amylases was found to be optimum as the fermentation was carried out at 37°C (Table 1). Further increase in the temperature gave insignificant production which might be due to the very sensitiveness of the organism to temperature [3].

The pH ranges from 6.0 to 7.0 have been reported for normal growth and enzyme activity in* Bacillus *strain isolated from soil [37]. When pH is altered below or above the optimum, activity decreased due to denaturation of proteins [38]. It is evident from the results of the present study that amylase production by the* B*.* subtilis* BI19 is better at neutral to alkaline range of pH (Table 1). The production was found to be optimum (7.77 U/mL/min) when the initial pH of the fermentation medium was maintained at 8.0. Further increase or decrease in pH resulted in gradual reduction of amylase production. These findings agree with those studies reported for* B*.* thermoleovorans* NP54 [39],* B*.* licheniformis* [40],* B*.* subtilis* JS-2004 [41], and* B*.* brevis* [42].

Optimization of incubation period was found to be very critical for maximum production of amylase [9]. In this study, the production of amylases was highest (8.67 U/mL/min) at 24 h after inoculation and decreased rapidly thereafter (Table 1). It might be due to that the organism entered in the stationary phase and fermentation approached its end point [43]. Maximum studies revealed that the production of amylase increased up to the level of 72 h of incubation [21]. Possible reason for amylase inactivation after 24 h might be due to release of high levels of intracellular proteases and/or secondary metabolites in the culture medium at the end of exponential phase. The present work is more encouraging as there was reduction in time period that can save energy requirement of the fermentation conditions and provide relatively efficient handling. Similar results were supported by Abate et al. (1999) [44].

Proper agitation is a basic need in order to achieve a good mixing, mass, and heat transfer in submerged fermentation [45]. In our study, amylase production was found to be increased steadily with the increase of agitation speed up to 150 rpm and the range of 140–170 rpm was found optimum (Table 1). Our findings are in good agreement with Nusrat and Rahman (2007) [21] and Sarikaya and Gürgün (2000) [18].

### 3.5. Effect of Volume of Fermentation Medium and Inoculums Size on Production of Amylase

Volume of fermentation medium plays critical roles in air and nutrient supply, growth of microorganisms, and production of enzyme [46]. In our study, maximum production was obtained at 50 mL of fermentation medium (Table 1). As the volume of the medium increased, the production was decreased most probably due to reduction in the agitation rate of medium that took place with high volume of fermentation medium leading to reduction in air supply as well as insufficient supply of nutrients required for biomass and enzyme synthesis [47].

Inoculum level reported to play critical role in submerged production [31]. In this investigation, production level was found to increase with increase in size of inoculum and found to be optimal at 2% (v/v) (Table 1). Further increase in the inoculum size greatly decreased the production that might be due to rapid growth of bacteria and rapid consumption of essential nutrient sources by bacteria in the initial stages. Malhotra et al. (2000) [39] also reported the similar findings.

### 3.6. Effect of Temperature on Activity and Stability of Crude Amylase

**Figure 5:**
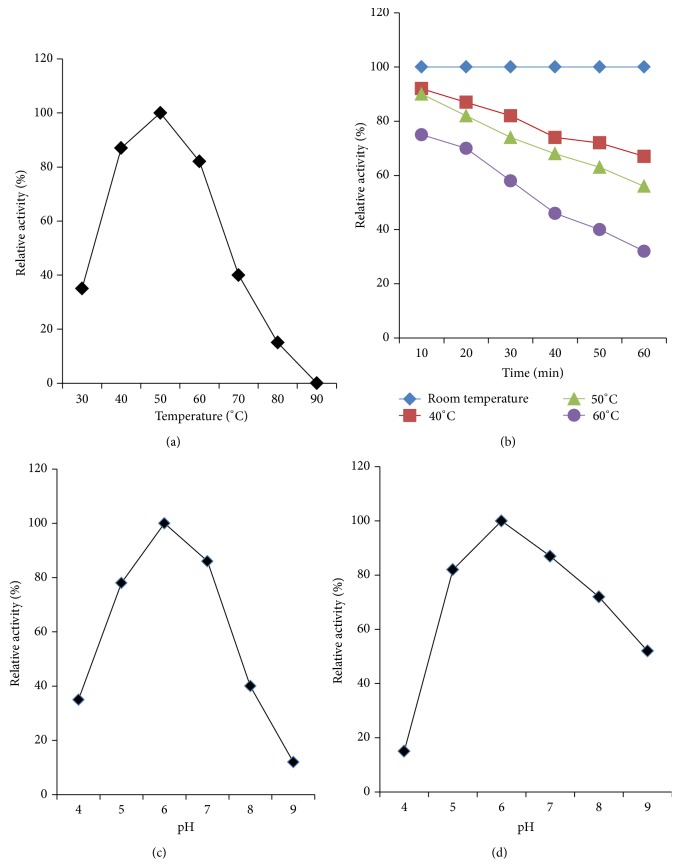
Partial characterization of crude amylase. (a) Effect of temperature on activity of crude amylase. (b) Effect of temperature on stability of crude amylase. (c) Effect of pH on activity of crude amylase. (d) Effect of pH on stability of crude amylase.

The effect of temperature on amylase activity was assayed at different temperatures ranging from 30 to 90°C at optimum pH 8.0. The optimum temperature for amylase activity was found to be between 40 and 60°C (Figure 5(a)). Amylase retained 100% relative activity when incubated at 50°C and, as temperature increased from 60 to 80°C, the activity was swiftly declined. At 80°C, the activity was the least (12%) and no activity was found at 90°C. These findings are comparable with that reported for the production of *α*-amylase using* B. amyloliquefaciens *by Demirkan (2011) [48]. In our study, crude amylase was heated at different temperatures for 1 h followed by testing its activity. The results showed that room temperature (25°C) was suitable for a long term stability of enzyme activity retaining 100% relative activity (Figure 5(b)). The enzyme retained above 60% relative activity even after heating at 50°C for 1 h. Thus these results concluded that the crude enzyme is moderately temperature stable. These findings agree with that reported by Yang and Liu, 2004 [49].

### 3.7. Effect of pH on Activity and Stability of Crude Amylase

A good industrial catalyst should be stable under the toughest operating conditions and for long durations [50]. When the crude amylase was treated at different pH, maximum activity was obtained at slightly acidic pH 6.0, retaining 100% activity as stated in Figure 5(c). There was a dramatic decline over activity when pH changes from 7.0 to 9.0 (retaining only 10% activity). The amylase retained more than 80% of its original activity between pH 5.0 and 7.0. The present study indicates that this enzyme prefers slightly acidic and/or alkaline pH for optimal activity. Similar preferred conditions for amylase activity have been found in previous studies [48]. Gupta et al. (2003) [51] stated this value as within the range of values for most starch degrading bacterial enzymes. The enzyme was found to be stable over a wide pH range (5.0 to 8.0) (Figure 5(d)). More than 60% residual activity was obtained at this range. From pH 4.0 to 6.0, there was a massive retention of about 60% activity. The possible reason may be the inhibition of enzyme active site by changing in the concentration of hydrogen ions.

## 4. Conclusion

Optimization of medium components and culture conditions for enhancing final production of amylase by* B. subtilis* BI19 and partial characterization of the crude amylase were carried out in this study. The most emerging and significant findings were the ability of this strain to utilize rice flour as sole carbon source to produce maximum level of amylase after only 24 h of incubation. Enzyme synthesis and secretion were affected greatly by addition of surfactants (positive inducer) and readily metabolizable glucose (negative inducer) in the basal medium. The optimum enzyme production by the bacterial isolate was found at 37°C, whereas maximum enzyme activity was found at 60°C and pH 5.0–7.0. Further research will be planned and carried out for improvement of the stain, purification of crude amylase, determination of encoded gene sequence of amylase, and further scaling up using fermenter.

## Figures and Tables

**Table 1:** Effect of different culture conditions for production of amylase by *Bacillus  subtilis *BI19 in shake-flask fermentation.

Culture conditions	Amylase activity (U/mL/min)	Relative activity (%)
Incubation temperature (°C)		
30	4.12 ± 0.31	56.82
32	5.59 ± 0.19	77.10
35	6.78 ± 0.94	93.51
37	7.25 ± 0.13	100.00
40	6.83 ± 0.45	94.20
42	5.20 ± 0.53	71.72
45	4.91 ± 0.45	67.72
Initial pH		
6.0	4.75 ± 0.47	52.90
6.5	5.37 ± 0.81	69.11
7.0	7.19 ± 0.19	92.53
7.5	7.51 ± 0.26	96.65
8.0	7.77 ± 0.10	100.00
8.5	6.60 ± 0.43	84.94
9.0	4.99 ± 0.53	64.22
Incubation period (h)		
12	3.61 ± 0.09	41.63
24	8.67 ± 0.08	100.00
36	7.44 ± 0.21	85.81
48	7.38 ± 0.14	85.12
60	7.29 ± 0.18	84.08
72	7.17 ± 0.07	82.70
Agitation speed (rpm)		
100	2.43 ± 0.23	33.20
120	3.94 ± 0.07	53.82
140	6.87 ± 0.22	93.85
150	7.32 ± 0.15	100.00
160	7.04 ± 0.17	96.17
170	6.73 ± 0.09	91.93
180	5.81 ± 0.23	79.37
Fermentation medium (mL)		
25	4.62 ± 0.52	63.55
50	7.27 ± 0.01	100.00
75	6.54 ± 0.19	89.96
100	6.04 ± 0.18	83.08
125	5.88 ± 0.07	80.88
150	5.09 ± 0.43	70.01
175	4.14 ± 0.31	56.95
Inoculum size (v/v %)		
1	5.98 ± 0.11	82.48
2	7.25 ± 0.61	100.00
3	7.00 ± 0.12	96.55
4	6.66 ± 0.19	91.86
5	4.27 ± 0.18	58.90

Data represent as mean for three replicates ± standard deviation.

## References

[1] Windish W. W., Mhatre N. S., Wayne W. U. (1995). Microbial amylases. *Advances in Applied Microbiology*.

[2] van der Maarel M. J. E. C., van der Veen B., Uitdehaag J. C. M., Leemhuis H., Dijkhuizen L. (2002). Properties and applications of starch-converting enzymes of the *α*-amylase family. *Journal of Biotechnology*.

[3] Pandey A., Nigam P., Soccol C. R., Soccol V. T., Singh D., Mohan R. (2000). Advances in microbial amylases. *Biotechnology and Applied Biochemistry*.

[4] Das K., Doley R., Mukherjee A. K. (2004). Purification and biochemical characterization of a thermostable, alkaliphilic, extracellular *α*-amylase from *Bacillus subtilis* DM-03, a strain isolated from the traditional fermented food of India. *Biotechnology and Applied Biochemistry*.

[5] Prakash B., Vidyasagar M., Madhukumar M. S., Muralikrishna G., Sreeramulu K. (2009). Production, purification, and characterization of two extremely halotolerant, thermostable, and alkali-stable *α*-amylases from *Chromohalobacter* sp. TVSP 101. *Process Biochemistry*.

[6] Azad M. A. K., Bae J.-H., Kim J.-S. (2009). Isolation and characterization of a novel thermostable *α*-amylase from Korean pine seeds. *New Biotechnology*.

[7] Burhan A., Nisa U., Gökhan C., Ömer C., Ashabil A., Osman G. (2003). Enzymatic properties of a novel thermostable, thermophilic, alkaline and chelator resistant amylase from an alkaliphilic *Bacillus* sp. isolate ANT-6. *Process Biochemistry*.

[8] Demirkan E. S., Mikami B., Adachi M., Higasa T., Utsumi S. (2005). *α*-Amylase from *B. amyloliquefaciens*: purification, characterization, raw starch degradation and expression in *E. coli*. *Process Biochemistry*.

[9] Deb P., Talukdar S. A., Mohsina K., Sarker P. K., Sayem S. M. A. (2013). Production and partial characterization of extracellular amylase enzyme from *Bacillus amyloliquefaciens* P-001. *SpringerPlus*.

[10] Gavrilescu M., Chisti Y. (2005). Biotechnology —a sustainable alternative for chemical industry. *Biotechnology Advances*.

[11] Atlas R. M., Parks L. C., Brown A. E. (1995). *Laboratory Manual of Experimental Microbiology*.

[12] Ball C., McGonagle M. P. (1978). Development and evaluation of a potency index screen for detecting mutants of *Penicillium chrysogenum* having increased penicillin yield. *Journal of Applied Bacteriology*.

[13] Hamilton L. M., Kelly C. T., Fogarty W. M. (1999). Production and properties of the raw starch-digesting *α*-amylase of *Bacillus* sp. IMD 435. *Process Biochemistry*.

[14] Rahman M. M., Basaglia M., Vendramin E. (2014). Bacterial diversity of a wooded riparian strip soil specifically designed for enhancing the denitrification process. *Biology and Fertility of Soils*.

[15] Rahman M. M., Basaglia M., Lorenzo F. (2014). A wooded riparian strip set up for nitrogen removal can affect the water flux microbial composition. *Italian Journal of Agronomy*.

[16] Marchesi J. R., Sato T., Weightman A. J. (1998). Design and evaluation of useful bacterium-specific PCR primers that amplify genes coding for bacterial 16S rRNA. *Applied and Environmental Microbiology*.

[17] Osborn A. M., Moore E. R. B., Timmis K. N. (2000). An evaluation of terminal-restriction fragment length polymorphism (T-RFLP) analysis for the study of microbial community structure and dynamics. *Environmental Microbiology*.

[18] Sarikaya E., Gürgün V. (2000). Increase of the alpha-amylase yield by some *Bacillus* strains. *Turkish Journal of Biology*.

[19] Gomes I., Sultana M., Uddin K., Gomes J., Steiner W., Gomes D. J. (2001). Nutrient composition and fermentation conditions for a-amylase production by *Bacillus amyloliquefaciens*. *Bangladesh Journal of Microbiology*.

[20] Miller G. L. (1959). Use of dinitrosalicylic acid reagent for determination of reducing sugar. *Analytical Chemistry*.

[21] Nusrat A., Rahman S. R. (2007). Comparative studies on the production of extracellular *α*-amylase by three mesophilic *Bacillus* isolates. *Bangladesh Journal of Microbiology*.

[22] Khajeh K., Naderi-Manesh H., Ranjbar B., Moosavi-Movahedi A. A., Nemat-Gorgani M. (2001). Chemical modification of lysine residues in *Bacillusα*-amylases: effect on activity and stability. *Enzyme and Microbial Technology*.

[23] Tonkova A., Ray R. C., Wards O. P. (2006). Microbial starch converting enzymes of the *α*-amylase family. *Microbial Biotechnology in Horticulture*.

[24] Kathiresan K., Manivannan S. (2006). *α*-amylase production by *Penicillium fellutanum* isolated from mangrove rhizosphere soil. *African Journal of Biotechnology*.

[25] Aiyer P. V. D. (2004). Effect of C:N ratio on alpha amylase production by *Bacillus licheniformis* SPT 27. *African Journal of Biotechnology*.

[26] Rukhaiyar R., Srivastava S. K. (1995). Effect of various carbon substrates on a-amylase production from *Bacillus* species. *World Journal of Microbiology and Biotechnology*.

[27] Sharma N., Vamil R., Ahmad S., Agarwal R. (2012). Effect of different carbon and nitrogen sources on *α*-amylase production from *Bacillus amyloliquefaciens*. *International Journal of Pharmaceutical Sciences and Research*.

[28] Hewitt C. J., Solomons G. L. (1996). The production of *α*-amylase (E.C.3.2.1.1.) by *Bacillus amyloliquefaciens*, in a complex and a totally defined synthetic culture medium. *Journal of Industrial Microbiology*.

[29] Bozic N., Ruiz J., Lopez-Santin J., Vujcic Z. (2011). Optimization of the growth and *α*-amylase production of *Bacillus subtilis* IP 5832 in shake flask and laboratory fermenter batch cultures. *Journal of the Serbian Chemical Society*.

[30] Swain M. R., Kar S., Padmaja G., Ray R. C. (2006). Partial characterization and optimization of production of extracellular *α*-amylase from B*acillus subtilis* isolated from culturable cow dung microflora. *Polish Journal of Microbiology*.

[31] Lin L. L., Chyau C. C., Hsu W. H. (1998). Production and properties of a raw-starch-degrading amylase from the thermophilic and alkaliphilic *Bacillus* sp. TS-23. *Biotechnology and Applied Biochemistry*.

[32] Arnesen S., Havn Eriksen S., Olsen J. Ø., Jensen B. (1998). Increased production of *α*-amylase from *Thermomyces lanuginosus* by the addition of Tween 80. *Enzyme and Microbial Technology*.

[33] Rao J. L. U. M., Satyanarayana T. (2003). Enhanced secretion and low temperature stabilization of a hyperthermostable and Ca^2+^-independent *α*-amylase of *Geobacillus thermoleovorans* by surfactants. *Letters in Applied Microbiology*.

[34] Moorthy S. N. (2002). Physicochemical and functional properties of tropical tuber starches: a review. *Starch/Staerke*.

[35] Palit S., Banerjee R. (2001). Optimization of extraction parameters for recovery of aamylase from the fermented bran of *Bacillus circulans* GRS313. *Brazilian Archives of Biology and Technology*.

[36] Mishra S., Noronha S. B., Suraishkumar G. K. (2005). Increase in enzyme productivity by induced oxidative stress in *Bacillus subtilis* cultures and analysis of its mechanism using microarray data. *Process Biochemistry*.

[37] Ramesh M. V., Lonsane B. K. (1991). Regulation of alpha-amylase production in *Bacillus licheniformis* M27 by enzyme end-products in submerged fermentation and its overcoming in solid state fermentation system. *Biotechnology Letters*.

[38] Hayashi T., Abiba T., Horikosh K. (1988). Properties of a new alkaline maltohexose forming amylases. *Applied Microbiology and Biotechnology*.

[39] Malhotra R., Noorwez S. M., Satyanarayana T. (2000). Production and partial characterization of thermostable and calcium-independent *α*-amylase of an extreme thermophile *Bacillus thermooleovorans* NP54. *Letters in Applied Microbiology*.

[40] Krishnan T., Chandra A. K. (1983). Purification and characterization of *α*-amylase from *Bacillus licheniformis* CUMC305. *Applied and Environmental Microbiology*.

[41] Asgher M., Asad M. J., Rahman S. U., Legge R. L. (2007). A thermostable *α*-amylase from a moderately thermophilic *Bacillus subtilis* strain for starch processing. *Journal of Food Engineering*.

[42] Tsvetkov V. T., Emanuilova E. I. (1989). Purification and properties of heat stable *α*-amylase from *Bacillus brevis*. *Applied Microbiology and Biotechnology*.

[43] Hillier P., Wase D. A. J., Emery A. N., Solomons G. L. (1997). Instability of *α*-amylase production and morphological variation in continuous culture of *Bacillus amyloliquefaciens* is associated with plasmid loss. *Process Biochemistry*.

[44] Abate C. M., Castro G. R., Siñeriz F., Callieri D. A. S. (1999). Production of amylolytic enzymes by *Bacillus amyloliquefaciens* in pure culture and in co-culture with *Zymomonas mobilis*. *Biotechnology Letters*.

[45] Amadi O. C., Okolo B. N. (2013). Influence of stirrer speed on the morphology of *Aspergillus carbonarius* var (Bainier) Thom IMI 366159 during raw starch digesting amylase production. *Asian Journal of Biological Sciences*.

[46] Ivanova V., Yankov D., Kabaivanova L., Pashkkoulov D. (2001). Simultaneous biosynthesis and purification of two extra cellular *Bacillus* hydrolases in aqueous two alpha amylases. *Journal of Biochemical Engineering*.

[47] Narang S., Satyanarayana T. (2001). Thermostable alpha-amylase production by an extreme thermophile Bacillus thermooleovorans. *Letters in Applied Microbiology*.

[48] Demirkan E. (2011). Production, purification, and characterization of *α*-amylase by *Bacillus subtilis* and its mutant derivates. *Turkish Journal of Biology*.

[49] Yang C.-H., Liu W.-H. (2004). Purification and properties of a maltotriose-producing *α*-amylase from *Thermobifida fusca*. *Enzyme and Microbial Technology*.

[50] Pal A., Khanum F. (2010). Production and extraction optimization of xylanase from *Aspergillus niger* DFR-5 through solid-state-fermentation. *Bioresource Technology*.

[51] Gupta R., Gigras P., Mohapatra H., Goswami V. K., Chauhan B. (2003). Microbial *α*-amylases: a biotechnological perspective. *Process Biochemistry*.

